# Supplementary material for: An open-source interactive AI framework for assisting automatic literature review in forensic medicine: Focus on brain injury mechanisms
Source: PLoS One. 2025 Aug 1;20(8):e0329349. doi: 10.1371/journal.pone.0329349 (PMC12316232; doi:10.1371/journal.pone.0329349)
Supplement: Attachment — Submitted filename: Response to reviewers.docx [file pone.0329349.s002.docx]

Dear editors and reviewers,

Thank you very much for your careful review and constructive comments on our manuscript titled “An Open-Source Interactive AI Framework for Assisting Automatic Literature Review in Forensic Medicine: Focus on Brain Injury Mechanisms”(PONE-D-24-60438). We truly appreciate the time and effort you have invested in evaluating our work. Your feedback has been invaluable in helping us improve the quality and clarity of our research.

We have carefully considered each of the reviewers' comments and have made the corresponding revisions to our manuscript. Below, we provide a detailed point-by-point response to the editors’ requirements and reviewers' comments, along with the changes we have implemented.

**Journal requirements:**

1. Please ensure that your manuscript meets PLOS ONE's style requirements, including those for file naming. The PLOS ONE style templates can be found at

https://journals.plos.org/plosone/s/file?id=wjVg/PLOSOne_formatting_sample_main_body.pdf and

https://journals.plos.org/plosone/s/file?id=ba62/PLOSOne_formatting_sample_title_authors_affiliations.pdf.

A1:

Thank you very much for your attention to our manuscript and your valuable comments. We have now revised the manuscript format in accordance with the journal's requirements. The changes have been carefully implemented to ensure compliance with the submission guidelines.

2. Please note that PLOS ONE has specific guidelines on code sharing for submissions in which author-generated code underpins the findings in the manuscript. In these cases, we expect all author-generated code to be made available without restrictions upon publication of the work. Please review our guidelines at https://journals.plos.org/plosone/s/materials-and-software-sharing#loc-sharing-code and ensure that your code is shared in a way that follows best practice and facilitates reproducibility and reuse.

A2:

Thank you very much for your attention to our manuscript and your valuable comments. We would like to clarify that our experiments did not involve any modifications to the ASReview code. The original code used in our study can be obtained directly from the authors of the ASReview model.

3. PLOS requires an ORCID iD for the corresponding author in Editorial Manager on papers submitted after December 6th, 2016. Please ensure that you have an ORCID iD and that it is validated in Editorial Manager. To do this, go to ‘Update my Information’ (in the upper left-hand corner of the main menu), and click on the Fetch/Validate link next to the ORCID field. This will take you to the ORCID site and allow you to create a new iD or authenticate a pre-existing iD in Editorial Manager.

A3:

Thank you very much for your attention to our manuscript and your valuable comments. We have updated the Corresponding Author's OCR ID in the system as required by the journal. The ID number is 0000-0002-2025-3254.

4. Thank you for stating the following financial disclosure:

 [The study was financially supported by grants from the Central Research Institute Public Project (GY2024D-1, GY2024Z-1), the National Natural Science Foundation of China (82171872), Shanghai Yangfan Special Programme (23YF1448700), Shanghai Key Laboratory of Forensic Medicine (21DZ2270800), Shanghai Forensic Service Platform, Key Laboratory of Forensic Science, Ministry of Justice, the Project of Shanghai Association of Forensic Science (SHSFJD2023-008), the 2023 Open Foundation of Key Laboratory of Forensic Pathology, Ministry of Public Security, P. R. China (GAFYBL202308).].

Please state what role the funders took in the study.  If the funders had no role, please state: ""The funders had no role in study design, data collection and analysis, decision to publish, or preparation of the manuscript.""

If this statement is not correct you must amend it as needed.

Please include this amended Role of Funder statement in your cover letter; we will change the online submission form on your behalf.

A4:

Thank you for your valuable comments regarding the funding information. We have carefully revised and streamlined the funding details in accordance with the journal's requirements. The revised section now includes the name of the funding agency, the specific title of the funding project, the project number, and a clear description of the specific purposes for which the funding was utilized. This revision aims to enhance transparency regarding the financial support for our research and to meet the expectations of both the journal and the funding institutions. We believe these changes address your concerns and provide a clear and comprehensive overview of the funding sources for our study.

5. In the online submission form, you indicated that [The data generated and analyzed during the study are available from the corresponding authors on reasonable request.].

All PLOS journals now require all data underlying the findings described in their manuscript to be freely available to other researchers, either 1. In a public repository, 2. Within the manuscript itself, or 3. Uploaded as supplementary information.

This policy applies to all data except where public deposition would breach compliance with the protocol approved by your research ethics board. If your data cannot be made publicly available for ethical or legal reasons (e.g., public availability would compromise patient privacy), please explain your reasons on resubmission and your exemption request will be escalated for approval.

A5:

Thank you very much for your attention to our manuscript and your valuable comments. We have revised the Data Availability Statements in accordance with the editor’s requirements. As our data do not involve ethical concerns or patient privacy issues, we declare that all data underlying the findings described in our manuscript are freely accessible to other researchers. The corresponding data are available within the manuscript itself. This complies with the editor’s requirements for data transparency.

**Reviewers' comments:**

Reviewer #1:

1) A sentence describing ASReview should be included in the abstract.

A1):

Thank you for your suggestion. We have incorporated a description of ASReview at the beginning of the abstract to provide readers with a clearer understanding of the model's functionality and the rationale behind our choice of this model. We believe this addition will enhance the readability and comprehension of our study.

2) The full article needs a grammar check. Especially the tasks/methods already performed or the conclusions drawn should be in the past tense. There are several instances where there is inconsistent use of present and past tense. I recommend rectifying this for better flow and good writing.

A2):

Thank you for your valuable feedback regarding the consistency of verb tenses in the manuscript. We have carefully reviewed the text and made the necessary adjustments to ensure that completed tasks, methods, and conclusions are consistently presented in the past tense. This revision aims to improve the overall coherence and clarity of the writing. We appreciate your attention to detail and believe these changes will enhance the quality of our manuscript.

3) Spacing after '.' at the end of sentences is inconsistent. There are also a few occurrences of additional spaces between words. Lastly, references are sometimes included after '.' and sometimes before. Please check the whole article for consistent formatting. Was this an issue with how the submission by the journal was formatted or in the original draft from the authors?

A3):

Thank you for your valuable feedback. We have now completed the corresponding revisions.

4) The introduction section can be shortened to make it concise.

A4):

Thank you for your insightful suggestion. We have carefully revised the Introduction section to enhance its accuracy and conciseness while preserving the original meaning. We believe these changes improve the clarity and effectiveness of the narrative.

5) The section "Data and models" should have model instead of models in the name as only one model is discussed in this article. Can there be a better worded heading instead of "Data and model.”

A5):

Thank you for your constructive suggestion. In this manuscript, the section in question primarily covers data collection and cleaning, gold standard establishment, and model parameter settings. Based on your feedback, we have revised the section title to "Data Preparation and Model Configuration". Specifically, "Data Preparation" now encompasses both data collection and cleaning, as well as the establishment of the gold standard, while “Model Configuration" is used to emphasize the process of model parameter settings. We believe these changes enhance the clarity and coherence of the section.

6) Data collection and filtering -

a. Check the word pertaining.

A6) a:

Thank you for your suggestion. We have carefully reviewed and corrected any spelling errors throughout the manuscript to ensure accuracy and consistency.

b. Zotero can include a reference, the version number, and the company name. This should be sufficient and the small section "Zotero 6.0.37" under "Deep learning model and settings" can be removed.

A6) b:

Thank you for your suggestion. We have added the version number of Zotero to this section and have removed the redundant content related to Zotero in the subsection titled "Deep Learning Model and Settings." This ensures clarity and avoids repetition in our manuscript.

7) Gold Standard -

a. It is a little misleading to call it dataset 1 and dataset 2 as they use the same data and just different truths (gold standard). Either change it to truth 1 and truth 2 or have an explicit sentence under this section clarifying that the data was the same and the truth was different.

A7) a:

Thank you for your suggestion. In fact, Dataset 1 and Dataset 2 represent two different processing approaches applied to the same portion of data, namely Gold Standard 1 and Gold Standard 2. To enhance clarity, as per your suggestion, we have renamed Dataset 1 to Truth 1 and Dataset 2 to Truth 2.

b. Three senior forensic experts were used to prepare the truths. Did they have craniocerebral injury and deep learning knowledge to handle this interdisciplinary review? Are these three experts only the "specialists" mentioned in the article? If yes, either use experts or specialists throughout.

c. Did the same three specialists categorize the 70 relevant papers into the five levels?

A7) b and c:

Thank you for your insightful comments regarding points b) and c) in section 7. We have now completed the corresponding revisions in the manuscript. To clarify, the three experts (referred to as "expert" or "specialist") mentioned in the manuscript are the same individuals throughout. These experts possess interdisciplinary expertise, enabling them to handle the intersection of traumatic brain injury and deep learning. They were responsible for annotating all 329 articles in the experiment and categorizing the 70 relevant papers into the five levels. To better convey this information, we have adopted your suggestion and consistently used the term "three experts" throughout the text. Additionally, we have dedicated a specific section to elaborate on their roles and qualifications.

8) Is it van de Schoot or Van De Schoot? Both are used in the article and since it is a proper noun, only one can be correct. Please correct this.

A8):

Thank you for your suggestion. We have corrected the scholar's name to Rens van de Schoot and have completed the revision in the manuscript.

9) Deep learning model and settings -

a. Shouldn't learning have capitalized L in the heading "Active learning for Systematic Reviews toolbox" heading?

A9) a:

Thank you for your suggestion. The manuscript has now been revised accordingly.

b. "The selection of these default values is justified by their consistently high performance across several benchmark experiments on various datasets." - Please add references to the benchmark experiments for this sentence showing the use of these default values.

A9) b:

We have provided a more detailed description of this section and added references to support the performance of the default parameters.

c. It is also mentioned that the default settings have short computation time - recommend including the actual time taken on average - can also be a range of time taken.

A9) c:

We have gathered extensive empirical evidence from the literature to support the efficiency of our default settings. Specifically, the combination of Naive Bayes (NB) and TF-IDF has been shown to demonstrate a relatively short average computation time across various datasets, typically ranging from a few seconds to tens of seconds, depending on the size and complexity of the dataset. For instance, van Dinter et al. , Marshall et al. , and Nagtegaal et al. have all confirmed that this combination is highly efficient in terms of computation time. Additionally, Ferdinands et al. and van de Schoot et al. have conducted experiments on multiple datasets with varying structures, sizes, and domains, and their results indicate that the NB + TF-IDF combination requires approximately 10 seconds to process a dataset of 1,847 documents and about 20 seconds to process a dataset of 8,896 documents[1][2].However, it is important to note that the findings by Ferdinands et al. and van de Schoot et al. have not yet been published in peer-reviewed journals. Therefore, we have not included formal citations for their work in this manuscript. Despite this, their contributions are acknowledged and have been instrumental in shaping our understanding of the computational efficiency of the NB + TF-IDF combination.

[1] Ferdinands, G., Schram, R., de Bruin, J., Bagheri, A., Oberski, D. L., Tummers, L. G., & van de Schoot, R. Active learning for screening prioritization in systematic reviews - A simulation study. Zenodo, 2020.

[2] van de Schoot, R., de Bruin, J., Schram, R., Zahedi, P., Kramer, B., Ferdinands, G., et al. ASReview: Active Learning for Systematic Reviews. Zenodo, 2020.

10) Methods - Stratified 5-fold cross-validation

a. Reword the last paragraph in this paragraph as the sentences seem redundant.

A10) a:

We have streamlined the last paragraph of the “Methods - Stratified 5-fold cross-validation” section to more clearly present the experimental steps.

b. Accuracy is not an ideal measure as the dataset is imbalanced. Why was AUC from the ROC curve not used as a performance metric given that it is not dependent on prevalence? Recommend reporting the AUC metric for all analyses reported in this article.

A10) b:

Thank you for your valuable comments. In response to your query, we have constructed the ROC curves and calculated the AUC based on the results of stratified five-fold cross-validation. The mean AUC of the ROC curve reached 0.98, which further demonstrates the robust performance of ASReview in predicting the relevance of forensic articles. These results have been incorporated into the revised manuscript (see Figure 4 and the corresponding discussion).

11) Does "encoding order" actually mean the sequential numbering of the 329 documents mentioned in the "Data collection and filtering" section? If yes, please specify this clearly under the data collection section rather than using it directly at the end of Methods. Also, can another phrase such as indexing or similar be used since "encoder" in AI means different?

A11):

Thank you for your insightful comments. We have revised the manuscript to address your concerns regarding the term "encoding order." Specifically, we have clarified that the term refers to the unique index numbers assigned to each entry in the dataset based on the order of collection and filtering. This clarification has been added to the "Data Collection and Filtering" section to ensure transparency and avoid confusion.

Additionally, we have replaced the term "encoding order" with "index order" throughout the manuscript to avoid any potential ambiguity with the term "encoder" in the context of artificial intelligence. This change ensures that our terminology is precise and aligns with standard academic practices.

We appreciate your attention to these details and believe that these revisions enhance the clarity and accuracy of our manuscript.

12) Is there other literature showing the use of the "snowballing" approach? If yes, please reference them with "snowballing" in the article.

A12):

Thank you for your valuable comments regarding the use of the term "snowballing" in our manuscript. We have revised the manuscript to replace the term "snowballing" with the more precise term "incremental learning approach". This change reflects the machine learning concept of iteratively building upon previous training sessions, which is more appropriate for describing our methodology in the context of our study.

13) A minor suggestion is to avoid using words such as demystify/admirably and keep them more technical or appropriate for scientific writing.

A13):

Thank you for your minor suggestion regarding the use of words such as "demystify" and "admirably." We have carefully revised the manuscript to ensure that the language is more technical and appropriate for scientific writing.

Specifically, we have made the following changes:

1.We have replaced the term "demystify" with "elucidate the underlying mechanisms" to provide a more technical and precise description of our objective in the experimental segment.

2.We have reviewed the entire manuscript to ensure that all language is consistent with scientific writing standards. Any non-technical expressions have been replaced with more appropriate terminology.

These changes aim to enhance the clarity and technical accuracy of our manuscript, ensuring that it aligns with the expectations of scientific writing.We believe these revisions have improved the overall quality of our work.

14) Comparative Analysis of Human and Machine Learning Scoring

a. Please clarify in the article that when the selection proportion was increased by 5% randomly for "Comparative Analysis of Human and Machine Learning Scoring," it was ensured that the previously selected data was retained and not selected again newly in the following iterations.

b. Also clarify ground truth for this - explaining that levels 1 to 3 of the experts were taken as relevant and levels 4 and 5 were taken as irrelevant.

A14):

Thank you again for your valuable feedback. We have revised the relevant descriptions to address the issues raised. The following clarifications are provided for your reference:

a. Incremental Learning Method

In fact, the incremental learning method (snowballing) described in the section "Methods—Assessing Model Robustness in Unbalanced Data Scenarios" was still employed here. Specifically, each extraction was based on the previous one, with an additional 5% of the total data added each time. This ensured that each new training set fully encompassed the content of the preceding training set. Consequently, each subsequent machine learning iteration was built upon the foundation of the previous iteration, rather than initiating a new learning session. This incremental learning approach effectively controlled variables and maintained consistency throughout the learning process.

However, given the necessity to include both relevant and irrelevant data in the training set, we divided the entire data of Truth 2 into two subsets: one relevant and one irrelevant. The relevant subset was derived from the results of a five-level scoring system applied by three experts, in accordance with the gold standard criteria. Subsequently, we proportionally sampled the training set from both subsets simultaneously. The phrase "instead of extracting articles in the order of their encoding" refers to the fact that we did not extract data sequentially based on the index order of the entire dataset. Instead, we sampled proportionally from the two subsets.

b. Expert Scoring Criteria

The expert scoring criteria have been detailed in the section "Data Preparation and Model Configuration—Gold Standard," with Table 1 provided for reference. We designed this scoring system to simulate a real-world retrieval process. Given the retrieval target of "deep learning" and "mechanisms of traumatic brain injury," which encompasses multiple core concepts and keywords, the following levels were established:

Level 1: Articles that fully meet all core concepts.

Level 3: Articles that meet some core concepts.

Level 5: Articles that do not directly meet the core concepts but have valuable experimental methods or ideas worth referring to.

Additionally, Levels 2 and 4 were introduced to address cases where experts were uncertain and found it difficult to classify the articles between two levels.

To resolve the ambiguity caused by the previous expression, we have thoroughly rewritten the entire section "Methods—Comparative Analysis of Human and Machine Learning Scoring." We welcome further suggestions to enhance clarity and accuracy.

15) "model's superior capability" under the Stratified 5-fold cross-validation section of Results should avoid using the word superior since there was no statistical test performed to show its superiority during 5-fold.

A15):

Thank you for your valuable suggestions. We have now completed the revisions in the corresponding sections of our manuscript.

16) Were the p-values adjusted for multiple comparisons? If yes, please specify and if no, justify why not.

A16):

In response to your query, we provide the following clarification: No p-value adjustment was performed in this study. In the analysis of data presented in the section "Assessing Model Robustness in Unbalanced Data Scenarios," we compared accuracy, precision, recall, F1 score, and specificity separately between two sets of experiments. These comparisons were conducted independently for each metric, without involving joint hypothesis testing across multiple parameters or multiple group comparisons. Therefore, this scenario does not constitute multiple comparisons. According to statistical principles, p-value adjustment is primarily used to control the false positive rate in the context of multiple comparisons (e.g., family-wise error rate or false discovery rate). Since no multiple comparisons were conducted in this study, p-value adjustment was deemed unnecessary.

17) "The Spearman correlation analysis revealed a positive correlation between human expectations (Table 6)." It will not be between human expectations; it will be with human expectations. Please check for grammar throughout.

A17):

Thank you for your suggestion. We have completed the revision in the relevant section and have also checked the grammar throughout the manuscript.

18) Suggestion to include future scope in the discussion section.

A18):

Thank you for your suggestion. We have incorporated the content into the Discussion section as recommended.

We kindly request that you consider our submission for publication and look forward to your positive response. Should you require any further information or clarification, please feel free to contact us.

Thank you for your time and consideration. We eagerly await your feedback.

Sincerely,

Corresponding Authors

Zhi-Ling Tian & Ning-Guo Liu

Email: tianzl@ssfjd.cn; [Liung@ssfjd.cn](mailto:Liung@ssfjd.cn)

Academy of Forensic Science, Shanghai Key Laboratory of Forensic Medicine, Shanghai Forensic Service Platform, Key Laboratory of Forensic Medicine, Ministry of Justice,1347# West Guangfu Road, Shanghai, 200063, People’s Republic of China.
