# Supplementary material for: An open-source interactive AI framework for assisting automatic literature review in forensic medicine: Focus on brain injury mechanisms
Source: PLoS One. 2025 Aug 1;20(8):e0329349. doi: 10.1371/journal.pone.0329349 (PMC12316232; doi:10.1371/journal.pone.0329349)
Supplement: Attachment — Submitted filename: Response_to_reviewers_auresp_2.docx [file pone.0329349.s003.docx]

Dear Editors,

PLOS ONE

May 25, 2025,

Thank you very much for your careful review and constructive comments on our manuscript titled "**An Open-Source Interactive AI Framework for Assisting Automatic Literature Review in Forensic Medicine: Focus on Brain Injury Mechanisms (PONE-D-24-60438)**". We truly appreciate the time and effort you have invested in evaluating our work. Your feedback has been invaluable in helping us improve the quality and clarity of our research.

We have carefully considered each of the reviewers' comments and have made the corresponding revisions to our manuscript. Below, we provide a detailed point-by-point response to the editors’ requirements and reviewers' comments, along with the changes we have implemented.

Response to ACADEMIC EDITOR:

1. address all comments remarked by reviewers

A1:

Thank you for your question and comments.

The revisions have been completed in response to the reviewer's request.

2. point out how you chose the state-of-the-art references in your manuscript

A2:

Thank you for your question and comments.

We have carefully revised the relevant descriptions in the manuscript as follows:

1. This study aims to explore the feasibility of applying active learning models to forensic medical literature retrieval. Given this objective, PubMed was selected as the retrieval platform. As a retrieval platform based on the US National Library of Medicine's MEDLINE database, PubMed's extensive and systematic medical literature collection system provides high-quality resources for global medical researchers, promoting and validating medical research.

2. Due to experimental needs, we chose the keyword combinations “deep learning and brain injury” and “deep learning and head injury” for reverse-chronological retrieval to collect all the literature under both combinations. Reverse-chronological retrieval was adopted to avoid the influence of PubMed's inherent algorithms, such as the “best match” strategy, which might bias the literature recommendations. Collecting all the literature under the keyword combinations aims to prevent subjective selection bias.

Based the strategy above, there were 419 articles in total under the two combinations, which were all collected. Then, following our screening criteria, we cleaned the data and obtained 329 valid articles.

The **problematic papers** were as follows:

1. Retracted articles: Including those retracted by authors and editors, which can be identified by retraction marks or statements on PubMed.

2. Special-format documents: Only literature from journal papers, conferences, and books was included.

3. Duplicated publications.

4. Duplicated collections: As two keyword combinations were used for retrieval, some articles might have been retrieved twice. For such cases, only one copy of the article was retained.

3. state what you mean by "reasonable request" for data accessibility

A3:

We have revised the data availability statement in accordance with PLOS ONE's requirements and uploaded the original data as supplementary material, ensuring unrestricted reader access to the study's raw data.

A3 is presented in '**Data Availability Statements**' on page 15:

All data underlying the findings described in our manuscript are freely accessible to other researchers.

Reviewer #1: All comments have been addressed by the authors appropriately. The paper can be accepted for publication as submitted.

A: Thank you for your review.

Your insightful suggestions have provided us with valuable guidance.

Reviewer #2: This manuscript presents an intriguing study that evaluates the performance of the machine learning tool ASReview for literature screening in the field of forensic science. Particularly noteworthy is the demonstration that artificial intelligence, specifically deep learning, can function effectively even in the complex and often scientifically ambiguous domain of forensic medicine. The observation that overfitting—akin to a human-like state of "overstudying" or absorbing excessive information—may actually degrade performance adds a novel and socially relevant dimension to the work, enhancing its originality and significance.

However, the abstract as currently written is somewhat difficult to follow, largely due to its use of technical jargon and abstract structure. This could obscure the central message of the study, especially for readers without a strong background in either medicine or data science. Therefore, I strongly recommend revising the abstract in a more accessible and structured manner, such as the following example:

Suggested Abstract (Structure and Example)

"In recent years, the explosive growth of academic publications has made it increasingly important to efficiently extract relevant information across all fields. This task is particularly challenging in forensic medicine, a discipline that requires interdisciplinary knowledge and frequently deals with scientifically inconclusive cases—placing a heavy burden on literature reviewers.

In response to this issue, the present study investigates whether ASReview, a machine learning-based automated literature screening tool, can be effectively applied to forensic science. As a simulated topic, we selected 'the application of deep learning in traumatic brain injury research' and analyzed 329 publications using stratified five-fold cross-validation.

The results showed that ASReview performed well, especially in automatically excluding irrelevant literature. When the proportion of labeled training data ranged from 10% to 55% of the entire dataset, the model’s output closely aligned with expert human judgment. However, when the proportion exceeded 80%, overfitting occurred, resulting in a decline in performance. This phenomenon—where the AI 'overlearns' and becomes less accurate—echoes real-world cognitive patterns in human learning and highlights both the promise and the limits of AI-assisted screening.

Overall, ASReview demonstrated practical utility even with limited training data, and it has the potential to significantly reduce the workload of researchers in forensic medicine—a field marked by complex and diverse literature."
A revision following this kind of structure—clearly stating the background, purpose, methods, results, and implications—would help make the abstract more compelling and understandable for a broader audience. I also suggest simplifying the overall writing style and minimizing the use of highly technical terminology where possible.

The study makes a valuable and empirical contribution by demonstrating the feasibility of AI-assisted literature review in the field of forensic science. I recommend acceptance pending revision, particularly with regard to improving the readability of the abstract and presentation of results.

Additionally, I strongly encourage the authors to revise the figure summarizing the five-tier relevance ranking to be more intuitive and immediately interpretable at a glance.

A:

Thank you for your question and comments.

We have revised the abstract into a structured format as per your suggestion to better serve reviewers and readers. However, due to the complexity of implementing comprehensive changes to the five-tier relevance ranking and potential mismatches with the original data, we have only made minor adjustments to the relevant descriptions. We would greatly appreciate it if you could agree that we will make no further modifications.

A is presented in '**Abstract**' on page 2:

**Background and Objective:** Systematic reviews and meta-analyses are critical in forensic medicine; however, these processes are labor-intensive and time-consuming. ASReview, an open-source machine learning framework, has demonstrated potential to improve the efficiency and transparency of systematic reviews in other disciplines. Nevertheless, its applicability to forensic medicine remains unexplored. This study evaluates the utility of ASReview for forensic medical literature review. **Methods:** A three-stage experimental design was implemented. First, stratified five-fold cross-validation was conducted to assess ASReview’s compatibility with forensic medical literature. Second, incremental learning and sampling methods were employed to analyze the model’s performance on imbalanced datasets and the effect of training set size on predictive accuracy. Third, gold standard were translated into computational languages to evaluate ASReview’s capacity to address real-world systematic review objectives. **Results:** ASReview exhibited robust viability for screening forensic medical literature. The tool efficiently prioritized relevant studies while excluding irrelevant records, thereby improving review productivity. Model performance remained stable when labeled training data constituted less than 80% of the total sample size. Notably, when the training set proportion ranged from 10% to 55%, ASReview’s predictions aligned closely with human reviewer decisions. **Conclusion:** ASReview represents a promising tool for forensic medical literature review. Its ability to handle imbalanced datasets and gather goal-oriented information enhances the efficiency and transparency of systematic reviews and meta-analyses in forensic medicine. Further research is required to optimize implementation strategies and validate its utility across diverse forensic medical contexts.

Reviewer #3: Writing:

1. There are numerous minor typos, missing characters and grammatical errors throughout manuscript.

A1:

Thank you for your question and comments.

We double-checked the manuscript totally and corrected minor typos, missing characters, and grammatical mistakes.

Methods:
In general, there seems to be a lack of rigor (blinding/masking, randomization, exclusion criteria, data availability etc) in the study design.

1. How were search results sorted in Pubmed (best match? most recent? author name? etc).

A1:

Thank you for your question and comments.

We chose the "**most recent**" strategy, which means retrieving articles in reverse chronological order of publication. This approach helps maintain data randomness. The "best match" option in PubMed uses algorithms to assign article weights, which may introduce bias. Similarly, criteria like author name or nationality could create bias. To avoid these potential biases in data collection, we specifically used the reverse order of publication dates as our search strategy.

2. Was search and retrieval automated or manual? If manual, were personnel blinded to author names, titles, journals etc. In what order were papers imported into Zotero?

A2:

Thank you for your question and comments.

**No manual selection was conducted.** As mentioned in A1, to ensure data randomness, we adopted the "most recent" search strategy, which retrieves articles in reverse chronological order of publication. Under the two keyword combinations of "deep learning and brain injury" and "deep learning and head injury", a total of 419 articles were retrieved using this strategy. **We downloaded all the articles and imported them into Zotero in the original chronological order from the search.** After importation, we did not use Zotero's built-in sorting options. This approach avoids the influence of Zotero's algorithms or programs on the data, thereby maximizing data randomization.

3. What additional steps, if any, were taken to avoid bias in the article listing order in Zotero?

A3:

Thank you for your question and comments.

In A1 and A2, we described several methods to prevent bias in data collection:

1. We selected the "most recent" search strategy, which retrieves articles in reverse chronological order of publication. This avoids bias from author information or search engine algorithms.

2. We downloaded all the literature retrieved from the two keyword combinations and imported it into Zotero, without any manual selection.

3. After importing the literature data into Zotero, we did not use any secondary sorting or processing options. This maintains the original chronological order of the data at the time of download, ensuring the randomness of the data.

4. How are "problematic papers" defined? The criteria should be clearly listed. Were these criteria determined prior to conducting the literature search?

A4:

Thank you for your question and comments.

"**Problematic papers**" is a placeholder term for the excluded papers. Before the data cleaning process, we established the following criteria for "problematic papers":

1. Retracted articles: Including those retracted by authors and editors, which can be identified by retraction marks or statements on PubMed.

2. Special-format documents: Only literature from journal papers, conferences, and books was included.

3. Duplicated publications.

4. Duplicated collections: As two keyword combinations were used for retrieval, some articles might have been retrieved twice. For such cases, only one copy of the article was retained.

Through screening and exclusion of these "problematic papers", we obtained 329 valid articles from the initial 419 retrieved. We hope our explanation meets your expectations, and we have revised the relevant parts in the manuscript.

A1 to A4 are presented in '**Data Preparation and Model Configuration-Data collection and filtering**' on page 4:

Based on our research topic of "the application of deep learning in traumatic brain injury research," we established search criteria to retrieve primary literature. Considering the characteristics of the forensic medicine field, PubMed was selected as the search platform. Due to the rapid increase in the number of scholarly articles pertaining to the application of deep learning in medical image analysis since 2015 [8-9], we queried PubMed [10] for papers containing "deep learning and brain injury" or "deep learning and head injury" in the title, keywords, or abstract from **January 1, 2014 to March 5, 2024** [11]. **We chose the "most recent" strategy,** which meant retrieving articles in reverse chronological order of publication. This approach helped maintain data randomness. The "best match" option in PubMed uses algorithms to assign article weights, which may introduce bias. Similarly, criteria like author name or nationality could create bias. A total of 419 articles were retrieved using this strategy. We downloaded all the articles (avoiding manual selection) and imported them into Zotero 6.0.37 in the original chronological order from the search. After importation, we did not use Zotero's sort and screen options. This approach avoided the influence of Zotero's algorithms or programs on the data, thereby maximizing data randomization.

Then we eliminated problematic papers. **Before the data cleaning process, we established the following criteria for "problematic papers":** 1. Retracted articles: These included articles retracted by authors and editors, which could be identified by retraction marks or statements on PubMed. 2. Special-format documents: Only literature from journal papers, conferences, and books was reserved. 3. Duplicated publications. 4. Duplicated collections: As two keyword combinations were used for retrieval, some articles might have been retrieved twice. For such cases, only one copy of the article was retained.

Ultimately, a total of 329 references were identified as valid. Each paper was assigned a unique index number in the order of collection and filtering, which is referred to as the "index order" throughout this study. This index order was used to systematically organize and reference the dataset during the experimental process.

5. Were ratings by three experts averaged or was there a tie-breaker?

A5:

Thank you for your question and comments.

We used the average score assigned by three experts.

6. Was inter-rater reliability calculated for the three expert reviewers?

A6:

Thank you for your question and comments.

We have added the relevant description to the 'Data Preparation and Model Configuration-Gold standard' section. In the experiment, there are two instances of expert-rating reliability assessment. First, when dividing the 329 articles into relevant and irrelevant categories, a Fleiss’ kappa value of 0.8548 was obtained. Second, for the five - level rating of the 70 relevant articles, the Fleiss’ kappa value was 0.827. These results indicate good consistency among the experts’ ratings, enabling their use as a gold standard. Notably, we required that a rating label be agreed upon by at least two experts to be valid. This approach further reduces potential discrepancies among the experts and improves the consistency of the labels.

A6 is presented in '**Data Preparation and Model Configuration-Gold standard**' on page 4：

In the gold standard, there were two assessments of expert-rating reliability. First, when the 329 articles were divided into relevant and irrelevant categories, a Fleiss’ kappa value of 0.85 was obtained. Second, for the five-level rating of the 70 relevant articles, the Fleiss’ kappa value was 0.83. These results indicated good consistency among the experts’ ratings, enabling their use as a gold standard. Notably, we required that a rating label be agreed upon by at least two experts to be valid. This approach further reduced potential discrepancies among the experts and improved the consistency of the labels.

7. Was dataset 1 shuffled randomly prior to splitting into 5 subsets for 5-fold CV?

A7:

Thank you for your question and comments.

As per the randomization protocols detailed in A1, A2, and A3, the data collection process inherently avoids subjectivity and selection bias. Hence, we did not perform any additional random shuffling.

8. Preparation of data (removal of "problematic papers", using Zotero's filtering capabilities etc) should be performed on the assigned training subset within each CV loop, rather than on the broader dataset1, to avoid data leakage.

A8:

Thank you for your question and comments.

Due to the small and imbalanced dataset, we prioritized data cleaning before grouping. This approach ensures each fold of the five - fold cross - validation has sufficient data. We've considered your suggested approach of grouping before cleaning. However, this method might lead to missing data within a group and cause unequal data distribution across the five folds of cross - validation.

9. The trained model should be prospectively assessed in a completely novel dataset that has not been previously curated by human experts. The resulting article classifications should then be reviewed by the human experts post-hoc to determine the model's real-world predictive performance.

A9:

Thank you for your question and comments.

We have added content on external validation to the Methods and Results sections. For the external validation set, we retrieved entirely new data using the "most recent" strategy with the keywords "deep learning and brain injury" and "deep learning and head injury". The training set covers from 2014 to March 5, 2024, while the external validation set includes all literature from March 6, 2024 to May 25, 2025, totaling 116 articles. After data cleaning, 109 valid articles were obtained.

When these 109 articles were input into the model for processing, three experts were also required to assess them. The experts categorized the 109 articles into 22 relevant and 87 irrelevant ones, with a Fleiss’ kappa value of 0.891. Comparing the model's output with the experts' ratings, we obtained an accuracy of 0.9266, precision of 0.7333, recall of 1.000, F1 score of 0.8461, and specificity of 0.9080. These results align with those of the test set, indicating the model's stable performance and its ability to accurately identify relevant literature and exclude irrelevant ones.

A9 is presented in '**Data Preparation and Model Configuration-External validation**' on page 6：

We collected entirely new data using the same procedures as those for the training set to **conduct external validation** of the model's performance. The training set covers from January 1, 2014 to March 5, 2024, while the external validation set includes all literature from March 6, 2024 to May 25, 2025, totaling 116 articles. After data cleaning, 109 valid articles were obtained.

In '**Methods**' on page 6：

Our experimental design was divided into three phases (Fig 1). Initially, we implemented stratified five-fold cross-validation to preliminarily evaluate the applicability of ASReview to forensic literature. If the results were positive, indicating that ASReview was suitable for the field of forensic science, we proceeded with the second and third experimental phases; if the results were negative, we would have reverted to the data collection and cleaning process. Subsequently, we employed an incremental learning sampling technique to investigate the model's performance on imbalanced datasets and the impact of training set size on its accuracy. Finally, we evaluated ASReview's capacity to meet real-world review expectations by translating reviewer requirements into computational language. **In addition, we selected entirely new data using the same procedures as those for the training set to conduct external validation of the model's performance.**

In '**Results--External validation**' on page 12：

**For the external validation set,** we retrieved entirely new data using the "most recent" strategy with the keywords "deep learning and brain injury" and "deep learning and head injury". The training set covers from January 1, 2014 to March 5, 2024, while the external validation set includes all literature from March 6, 2024 to May 25, 2025, totaling 116 articles. After data cleaning, 109 valid articles were obtained.

When we input these 109 articles into the model for processing, three experts assessed them. The experts categorized the 109 articles into 22 relevant and 87 irrelevant ones, with a Fleiss’ kappa value of 0.89. Comparing the model's output with the experts' ratings, we obtained an accuracy of 0.93, precision of 0.73, recall of 1.00, F1 score of 0.85, and specificity of 0.91. These results align with those of the test set, indicating the model's stable performance and its ability to accurately identify relevant literature and exclude irrelevant ones.

Data Availability:

1. Data is not fully available without restriction. Authors state the data are "available upon reasonable request". Who decides what a reasonable request is? This stance does not align with the authors' answer in the Data Availability Statement, and is contrary to PLOS Data policy.

A1:

Thank you for your question and comments.

We have revised the data availability statement in accordance with PLOS ONE's requirements and uploaded the original data as supplementary material, ensuring unrestricted reader access to the study's raw data.

A1 is presented in '**Data Availability Statements**' on page 15:

All data underlying the findings described in our manuscript are freely accessible to other researchers.

Thank you for your time and consideration. We eagerly await your feedback.

Sincerely,

Corresponding Authors

Zhi-Ling Tian & Ning-Guo Liu

Email: tianzl@ssfjd.cn; [Liung@ssfjd.cn](mailto:Liung@ssfjd.cn)

Academy of Forensic Science, Shanghai Key Laboratory of Forensic Medicine, Shanghai Forensic Service Platform, Key Laboratory of Forensic Medicine, Ministry of Justice,1347# West Guangfu Road, Shanghai, 200063, People’s Republic of China.
