# Supplementary material for: An open-source interactive AI framework for assisting automatic literature review in forensic medicine: Focus on brain injury mechanisms
Source: PLoS One. 2025 Aug 1;20(8):e0329349. doi: 10.1371/journal.pone.0329349 (PMC12316232; doi:10.1371/journal.pone.0329349)
Supplement: Attachment — Submitted filename: Response_to_reviewers_auresp_3.docx [file pone.0329349.s004.docx]

Dear Reviewers,

PLOS ONE

July 10, 2025,

Thank you very much for your careful review and constructive comments on our manuscript titled "**An Open-Source Interactive AI Framework for Assisting Automatic Literature Review in Forensic Medicine: Focus on Brain Injury Mechanisms (PONE-D-24-60438)**". We truly appreciate the time and effort you have invested in evaluating our work. Your feedback has been invaluable in helping us improve the quality and clarity of our research.

We have carefully considered each of the reviewers' comments and have made the corresponding revisions to our manuscript. Below, we provide a detailed point-by-point response to the editors’ requirements and reviewers' comments, along with the changes we have implemented.

**Reviewer #2**:

6. Review Comments to the Author

All comments have been addressed by the authors appropriately. The paper can be accepted for publication as submitted.

A6:

Thank you for your careful review and valuable comments regarding our manuscript.

Your insightful suggestions have provided us with valuable guidance.

**Reviewer #4**:

4. Have the authors made all data underlying the findings in their manuscript fully available?

The [PLOS Data policy](http://www.plosone.org/static/policies.action#sharing) requires authors to make all data underlying the findings described in their manuscript fully available without restriction, with rare exception (please refer to the Data Availability Statement in the manuscript PDF file). The data should be provided as part of the manuscript or its supporting information, or deposited to a public repository. For example, in addition to summary statistics, the data points behind means, medians and variance measures should be available. If there are restrictions on publicly sharing data—e.g. participant privacy or use of data from a third party—those must be specified.

A4:

Thank you for your question and comments.

In response to your suggestions, we have carefully reviewed the data associated with our manuscript that was previously uploaded to the PLOS ONE submission system. During the last revision, we included **the original literature information** as well as **the output results from our machine learning model**. In this revision, we will supplement **the data with the manual scoring results from three experts**. This additional information will further support the summary statistics and provide context for the data points underlying the means, medians, and variance measures. Please find the file named "**Experimental data.zip**" in the uploaded Supporting Information section.

We believe that these additions will enhance the robustness and transparency of our data analysis. Thank you again for your insightful feedback, which has been instrumental in improving our manuscript.

6. Review Comments to the Author

The manuscript is technically sound, and the presented data adequately support its conclusions—with a few caveats and suggestions for improvement. The statistical analysis appears to be appropriate and rigorous. I can’t confirm whether the data are fully available. If they’re not deposited in a public repository and documented in the manuscript, they haven’t met strong open-data standards. The manuscript is clearly organized and readable.

A6:

Thank you once again for your thorough review and valuable comments.

Your feedback has been instrumental in helping us gain a deeper understanding of the PLOS Data policy and further enhance the scientific rigor of our manuscript.

Thank you for your time and consideration. We eagerly await your feedback.

Sincerely,

Corresponding Authors

Zhi-Ling Tian & Ning-Guo Liu

Email: tianzl@ssfjd.cn; [Liung@ssfjd.cn](mailto:Liung@ssfjd.cn)

Academy of Forensic Science, Shanghai Key Laboratory of Forensic Medicine, Shanghai Forensic Service Platform, Key Laboratory of Forensic Medicine, Ministry of Justice,1347# West Guangfu Road, Shanghai, 200063, People’s Republic of China.
